# GC-MS Screening Analysis for the Identification of Potential Migrants in Plastic and Paper-Based Candy Wrappers

**DOI:** 10.3390/polym10070802

**Published:** 2018-07-21

**Authors:** Soraya Galmán Graíño, Raquel Sendón, Julia López Hernández, Ana Rodríguez-Bernaldo de Quirós

**Affiliations:** Department of Analytical Chemistry, Nutrition and Food Science, Faculty of Pharmacy, University of Santiago de Compostela, 15782-Santiago de Compostela, Spain; galman.graino@gmail.com (S.G.G.); raquel.sendon@usc.es (R.S.); julia.lopez.hernandez@usc.es (J.L.H.)

**Keywords:** GC-MS, non-targeted analysis, targeted analysis, photoinitiators, NIAS, candy wrappers

## Abstract

Food packaging materials may be a potential source of contamination through the migration of components from the material into foodstuffs. Potential migrants can be known substances such as additives (e.g., plasticizers, stabilizers, antioxidants, etc.), monomers, and so on. However, they can also be unknown substances, which could be non-intentionally added substances (NIAS). In the present study, non-targeted analysis using mass spectrometry coupled to gas chromatography (GC-MS) for the identification of migrants in plastic and paper-based candy wrappers was performed. Samples were analyzed after extraction with acetonitrile. Numerous compounds including *N*-alkanes, phthalates, acetyl tributyl citrate, tributyl aconitate, bis(2-ethylhexyl) adipate, butylated hydroxytoluene, etc. were identified. Many of the compounds detected in plastic samples are not included in the positive list of the authorized substances. One non-intentionally added substance, 7,9-Di-tert-butyl-1-oxaspiro(4,5)deca-6-9-diene-2,8-dione, which has been reported as a degradation product of the antioxidant Irganox 1010, was found in several samples of both plastic and paper packaging. The proposed method was shown to be a useful approach for the identification of potential migrants in packaging samples. The toxicity of the compounds identified was estimated according to Cramer rules. Then, a second targeted analysis was also conducted in order to identify photoinitiators; among the analyzed compounds, only 2-hydroxybenzophenone was found in five samples.

## 1. Introduction

Packaging has become essential since protecting packaged food from contamination facilitates their transport and increases their shelf life. However, the use of packaging introduces a safety concern because of the possible migration of dangerous substances from the packaging material into the foodstuffs [1,2,3]. Additives, components of the printing inks such as photoinitiators or residues of monomers are some of the substances of special interest from the food safety point of view. The additives such as plasticizers, slip agents, antioxidants, stabilizers, UV absorbers, etc. are used to improve the properties of the materials.

These substances of low molecular weight are of major concern, since they would be absorbed in the digestive tract, and could represent a risk to the health of the consumer. On the other hand, they can also produce changes in the organoleptic properties of the food.

Besides intentionally added substances, non-intentionally added substances (NIAS) including impurities of additives and monomers, degradation or reaction products, and neo-formed compounds may remain in the final product and consequently reach the packaged food [4,5].

All food contact materials and articles are regulated by the Framework Regulation (EC) No 1935/2004 [6]. The aforementioned regulation lays down the requirements that must be fulfilled by materials and articles that are intended to come into contact with food. In summary, under foreseeable conditions of use, they must not release components in quantities that represent a danger to human health, cause an unacceptable change in the composition of the food, or cause alterations in the organoleptic characteristics. In addition, labeling and advertising must not mislead consumers.

For plastic food contact materials, Regulation (EU) 10/2011 [7] includes the European Union positive list of monomers, additives, and starting substances allowed in the manufacture of plastic food contact materials and the restrictions of use, namely the specific migration limit (SML), overall migration, and limit of content of the substance in the material or article. This regulation also mentions the need to consider NIAS for risk assessments. Paper and cardboard are also widely used for food packaging applications, but the regulation for these materials is not harmonized at the community level.

The identification of potential migrants is the first step to evaluate the safety of food packaging materials. Mass spectrometry coupled to gas chromatography (GC-MS) for volatile and semi-volatile compounds or liquid chromatography (LC-MS) for non-volatile compounds appears as a useful and powerful technology for targeted and untargeted analysis.

In this work, a GC-MS non-targeted analysis for the identification of unknown potential migrants both intentionally and non-intentionally added substances in plastic and paper-based candy wrappers was carried out. A targeted analysis to identify specifically certain photoinitiators was also performed. The toxicity of the identified compounds was estimated by means of Cramer rules. The analysis of this type of packaging is of particular interest since the main consumers of sweets are children, who are a vulnerable group from the exposure point of view. Furthermore, the relationship food/food contact material is bigger than in other food products, which favors the migration.

## 2. Materials and Methods 

### 2.1. Reagents and Standards

Methanol, acetonitrile, n-hexane, and tetrahydrofuran liquid chromatography grade were provided from Merck KGaA (Darmstadt, Germany). Ultrapure water (Type I) was obtained with an Automatic Plus purification system (Wasserlab, Navarra, Spain). All of the analytical standards were from Sigma Aldrich (St. Louis, MO, USA). Acetyl tributyl citrate (≥98%) (CAS 77-90-7); benzyl butyl phthalate (≥98%) (CAS 85-68-7); butylated hydroxytoluene (≥99%) (CAS 128-37-0); bis(2-ethylhexyl) adipate (≥99%) (CAS 103-23-1); bis(2-ethylhexyl) phthalate (≥98%) (CAS 117-81-7); diethyl phthalate (≥99%) (CAS 84-66-2); diisobutyl phthalate (≥99%) (CAS 84-69-5); squalene (≥98%) (CAS 111-02-4); octadecane (≥99.9%) (CAS 593-45-3); triacetin (≥99%) (CAS 1029-76-1); glycerol tricaprylate (≥99%) (CAS 538-23-8). C7-C30 Saturated alkane mixture (1000 μg/mL each component in hexane).

Photoinitiators standards: 1-hydroxycyclohexyl-phenyl-ketone (99%) (CAS 947-19-3); 2,2-dimethoxy-2-phenyl acetophenone (99%) (CAS 24650-42-8); methyl-2-benzoylbenzoate (97%) (CAS 606-28-0); 2-hydroxybenzophenone (99%) (CAS 117-99-7); 4-methyl benzophenone (99%) (CAS 134-84-9); 4-phenyl benzophenone (99%) (CAS 2128-93-0); ethyl-4-(dimethylamino)benzoate (99%) (CAS 10287-53-3) were obtained from Aldrich (St. Louis, MO, USA) and benzophenone (99%) (CAS 119-61-9) was from Fluka (Stenheim, Switzerland).

Stock standard solutions were prepared in acetonitrile and hexane and stored at 4 °C until the analysis.

### 2.2. Samples and Extraction Procedure

Several candy wrappers based on plastic and paper materials and from different brands and two popcorn packages were analyzed in order to identify potential migrants. Table 1 summarized the samples analyzed in the study.

To extract the migrants, a known surface of the packaging material was put in contact with 25 mL of acetonitrile for 6 h at 70 °C. Then, the extracts were evaporated in a rotavapor system (Buchi, Postfach, Switzerland), and the residue was re-dissolved in a known volume of acetonitrile. An aliquot of the resulting solution was filtered through a 0.45 μm filter (Advanted, Toyo Roshi Kaisha, Ogawa-machi, Hiki-gun, Saitama, Japan) and injected into the GC-MS.

### 2.3. Equipment

#### GC-MS Analysis

##### Non-Targeted Analysis

A Thermo Scientific Trace 1300 Series gas chromatograph (Thermo Fisher Scientific, San José, CA, USA) with a Trace ISQ LT mass detector (San José, CA, USA) (GC-MS) equipped with a Thermo Scientific AI 1310 automatic injector (San José, CA, USA) was used to carry out the analysis. The chromatographic conditions were as follows: a ZB-5MS (30 m × 0.25 mm × 0.25 µm) column from Phenomenex^®^ (Torrance, CA, USA) was employed; the injector temperature was 300 °C. The injection mode was splitless, and the injection volume 1 μL. Helium was the carrier gas at a flow of 1 mL/min. The transfer line and source temperature were set at 300 °C. The oven temperature was initially at 40 °C for 2 min, then increased at a rate of 9 °C/min until 300 °C and held at 300 °C for 3 min. The chromatograms were acquired in full scan mode (*m*/*z* 35-500). The mass spectral libraries NIST/EPA/NIH 11 (version 2.0) and Wiley Registry TM 8th edition were used for identification purposes.

##### Targeted Analysis

Under the same chromatographic conditions, a targeted analysis to identify the photoinitiators in the extracts of the packaging samples was performed. The analyses were conducted using SIM mode.

## 3. Results and Discussion

### 3.1. Non-Targeted Analysis

#### 3.1.1. Plastic-Based Materials

A total of eight plastic candy wrappers were analyzed in order to identify potential migrants; for that purpose, a GC-MS non-targeted screening was carried out. 

Firstly, packaging samples were extracted, several extraction solvents and time-temperature conditions were tried (acetonitrile 6 h: 70 °C; acetonitrile 24 h: 70 °C; hexane 4 h: 60 °C; tetrahydrofuran 4 h: 60 °C and methanol 24 h: 70 °C) with the aim to achieve the most suitable extraction for the most compounds. It was observed that hexane extracted less analytes than the other solvents tested. Particularly, the compounds eluted in the first 20 min. On the other hand, THF and methanol showed a lower extraction efficiency compared with acetonitrile (ACN). When ACN was used as an extraction solvent, no important differences were observed regarding the extraction time. The best conditions turned out to be acetonitrile as extraction solvent at 70 °C for 6 h.

Twenty three volatile and semi-volatile compounds were identified. Eighteen compounds were positively confirmed by comparison with standards and the rest of the compounds were tentatively identified.

Cramer rules were applied in order to estimate the toxicity of the compounds identified. The software used was Toxtree v2.6.13 (Ideaconsult Ltd., Sofia, Bulgaria) (based on a decision tree approach) [8]. Substances are classified into three classes taking into account their chemical structures; thus, class I corresponds to low toxicity, and class II and class III correspond to intermediate and high toxicity, respectively [9].

**Figure 1:**
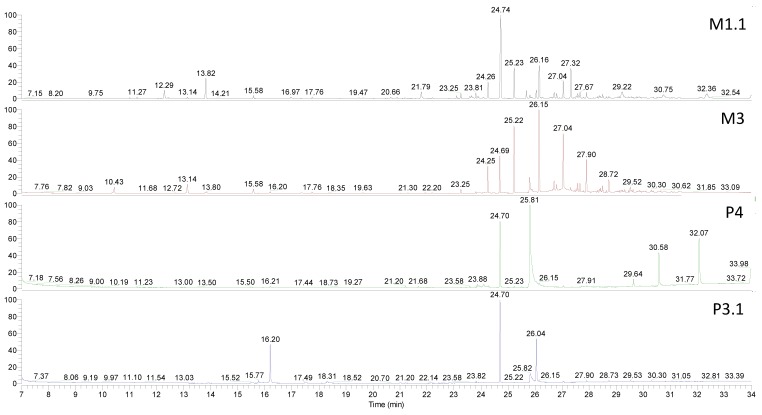
Mass spectrometry coupled to gas chromatography (GC-MS) chromatograms of samples M1.1, M3, P4, and P3.1.

Chromatograms corresponding to samples M1.1, M3, P4, and P3.1 are presented in Figure 1.

Table 2 summarizes the compounds identified in the acetonitrile extracts, but only library matches with a direct matching factor (SI) and a reverse search matching (RSI) higher than 700 were included.

N-alkanes between C21 and C29 were identified in most of the wrappers that were analyzed. Thus, pentacosane was found in all of samples, while, among others, tricosane, heptacosane and octacosane were found in seven of the eight samples analyzed (P1, P3.1, P3.2, P4, P5, P6 and P7). On the other hand, the sample P7 contained all of the identified alkanes except docosane. Saturated alkanes are usually present in polymers.

Rani et al. (2015) [10] reported the presence of hydrocarbons in plastic materials such as polypropylene (PP) and polyethylene (PE) and as well in polyethyl terephthalate (PET) and acrylic/styrene. They were also found in both virgin and recycled PP and in PP bottles [11,12].

Butylated hydroxytoluene (BHT) is a common antioxidant that is used as a stabilizer for polyolefins. It has also been identified in polyurethane adhesives [13,14]. Its use as an additive in the manufacture of plastic materials that are intended to come into contact with food is allowed, and has a specific migration limit (SML) of 3 mg/kg (Commission Regulation (EU) No 10/2011) [7]. Besides, it is also employed as a food additive. The antioxidant was identified in six samples (P2, P3.1, P3.2, P4, P5 and P6). Moreover, 2,6-Di-tert-butyl-4-methylene-2,5-cyclohexadienone, a degradation product of BHT, was detected in three samples (P3.1, P3.2 and P5). Particular attention has been paid to the degradation products since according to some studies, they could represent a risk for the consumers’ health [15]. Both compounds identified are classified as class II according to Cramer rules.

Different plasticizers such as acetyl tributyl citrate, tributyl aconitate, bis(2-ethylhexyl) adipate, and phthalates were also identified in the plastic wrappers analyzed. The main function of plasticizers is to improve the flexibility and processability of polymers [16,17,18]. Acetyl tributyl citrate and tributyl aconitate were identified in all of the samples, while bis(2-ethylhexyl) adipate was identified in all of the samples except in samples P1 and P7. Due to its low cost, this compound is widely used, especially in polyvinyl chloride (PVC) films [16,17,19]. Among the phthalates, diethyl phthalate and bis(2-ethylhexyl) phthalate were detected in all of the samples except in sample P2 and P3.1, respectively. Diisobutyl phthalate was identified in the samples P1, P3.2, P4 and P5. Low molecular weight phthalates such as diethyl phthalate and diisobutyl phthalate have been used, among other applications in printing inks, adhesives, etc. On the other hand, high molecular weight phthalates such as bis(2-ethylhexyl) phthalate are mostly used in PVC [20]. Some plasticizers, including bis(2-ethylhexyl) adipate and phthalates, have been described as endocrine disruptors [19,21].

Triacetin was detected only in sample P2; this substance has been reported as the main compound in plastic laminated paper [22]. Besides its use as food additive, it is allowed and has been classified as Generally Recognized As Safe (GRAS) by the Food and Drug Administration (FDA) [23].

7,9-Di-tert-butyl-1-oxaspiro(4,5)deca-6-9-diene-2,8-dione was the only identified compound classified in category III according to the Cramer rules. This substance has been described as a degradation product of the antioxidant Irganox 1010 [13,24]. Therefore, it could be considered as a non-intentionally added substance (NIAS) [4]. It was detected in all of the samples except in sample P4.

Diethyl maleate was detected in four samples (P1, P3.1, P3.2 and P.5); this compound is used to functionalize polymers such as polyolefins [25].

Other compounds that were also identified were squalene, a hydrocarbon used as plasticizer, which was detected in all of the samples, and glycerol tricaprylate, a lubricant found in samples P1, P6 and P7 [10,26].

It is important to note that many of the identified compounds are not on the European Union positive list of the allowed substances to be employed in plastic materials intended to come into contact with food.

#### 3.1.2. Paper-Based Materials

Table 3 list the 28 compounds identified in paper-based wrappers; 20 of them were confirmed with standards. Several of the compounds found such as, *N*-alkanes, phthalates, butylated hydroxytoluene, acetyl tributyl citrate, tributyl aconitate, bis(2-ethylhexyl) adipate, and so on were also identified in the plastic-based samples.

Regarding *N*-alkanes in these paper-based samples, we also identified octadecane, nonadecane, and eicosane with 18, 19 and 20 carbon atoms. Octadecane was detected in samples M1.1, M1.2, M3, M4 and M5; in the case of nonadecane and eicosane, they were found in all samples except in samples PA1 and PA2 nonadecane and sample PA2 eicosane. As it has been reported elsewhere, N-alkanes may have their origin in the paraffin wax, which is commonly used as coating in paper-based materials for food packaging applications [27].

With respect to phthalates, bis(2-ethylhexyl) phthalate was identified in all of the samples, whereas diethyl phthalate and diisobutyl phthalate were detected in all of the samples except in samples PA2 and M4, respectively. Benzyl butyl phthalate was only identified in three samples M1.1, M1.2, and M5, and was not found in plastic-based samples. Other plasticizers such as acetyl tributyl citrate was detected in all of the samples except in M1.2 or bis(2-ethylhexyl) adipate, which was identified in four samples: M1.1, M1.2, M3 and PA2.

Triacetin was identified in seven of the nine samples analyzed (M1.1, M1.2, M2.1, M2.2, M3, M4, and M5); however, in plastic-based materials, it was only detected in one sample.

Other compounds identified were heptadecylcyclohexane, octadecanoic acid, and palmitic acid, which have been reported as a lubricant, a slip agent, and a slip agent degradant, respectively [28].

7,9-Di-tert-butyl-1-oxaspiro(4,5)deca-6-9-diene-2,8-dione was identified in M1.1, M1.2, M2.1, M2.2, M3, M4 and M5; as it has been commented on above, it is classified in class III according to Cramer rules.

### 3.2. Targeted Analysis

With the aim to identify photoinitiators in the packaging samples, a target analysis was carried out. Only one of the photoinitiators evaluated was detected in the samples analyzed. 2-Hydroxybenzophenone was found in five samples: PA1, PA2, P2, P6 and P7.

In brief, here we present a simple methodology based on a GC-MS analysis to identify potential volatile migrants in both intentionally and non-intentionally added substances in food packaging materials. The proposed method could be useful as a screening approach in control laboratories for compliance with legislation. Our results showed that many compounds identified in plastic-based materials are not in the positive list of the Regulation 10/2011 [7].

## Figures and Tables

**Table 1:** Samples analyzed in the study.

Plastic Based Materials		Paper Based Materials	
Code	Sample description	Code	Sample description
P1	Strawberry candy brand 0	M1.1	Banana chewy candy brand A
P2	Strawberry lollipop brand 1	M1.2	Strawberry chewy candy brand A
P3.1	Cherry chewy candy brand 2	M2.1	Coke chewy candy brand B
P3.2	Strawberry chewy candy brand 2	M2.2	Lemon chewy candy brand B
P4	Strawberry lollipop brand 3	M.3	Strawberry chewy candy brand C
P5	Lemon candy brand 4	M.4	Blackberry candy brand D
P6	Lemon candy brand 5	M.5	Bilberry candy brand D
P7	Strawberry chewy candy brand 6	PA1	Popcorn brand E
		PA2	Popcorn brand F

**Table 2:** Compounds identified in the plastic-based candy wrappers and their class of toxicity according to Cramer rules.

*t*_R_ (min)	Compound	CAS	*m*/*z*	TC	SML	P1	P2	P3.1	P3.2	P4	P5	P6	P7
					Use: M/A								
11.07	Diethyl maleate	141-05-9	99, 127	I	_	X		X	X		X		
13.81	* Triacetin	102-76-1	43, 103, 145	I	_		X						
14.34	Propanoic acid, 2-methyl-, 3-Hydroxy-2,4,4-trimethylpentyl ester	74367-34-3	71, 56, 43, 89	II	_		X		X				
15.78	2,6-Di-tert-butyl-4-methylene-2,5-cyclohexadienone	2607-52-5	161, 203, 175	II				X	X		X		
16.21	* Butylated hydroxytoluene	128-37-0	205, 220	II	3A		X	X	X	X	X	X	
17.35	* Diethyl phthalate	84-66-2	149, 177	I	_	X		X	X	X	X	X	X
20.67	* Diisobutyl phthalate	84-69-5	149, 223	I	_	X			X	X	X		
21.19	7,9-Di-tert-butyl-1-oxaspiro(4,5)deca-6-9-diene-2,8-dione	82304-66-3	57, 205, 55, 175	III	_	X	X	X	X		X	X	X
23.26	* Heneicosane	629-94-7	57, 71, 85	I	_		X			X	X	X	X
23.82	Tributyl aconitate	7568-58-3	112, 157, 139	I	_	X	X	X	X	X	X	X	X
24.26	* Docosane	629-97-0	57, 71, 85	I	_		X	X		X	X	X	
24.71	* Acetyl tributyl citrate	77-90-7	185, 259, 129	I	** 60A	X	X	X	X	X	X	X	X
25.22	* Tricosane	638-67-5	57, 71, 85	I	_	X		X	X	X	X	X	X
26.06	* Bis(2-ethylhexyl) adipate	103-23-1	129, 147	I	** 60A		X	X	X	X	X	X	
26.15	* Tetracosane	646-31-1	57,71, 85	I	_	X	X		X	X	X	X	X
27.04	* Pentacosane	629-99-2	57,71, 85	I	_	X	X	X	X	X	X	X	X
27.32	* Bis(2-ethylhexyl) phthalate	117-81-7	149, 167	I	** 1,5A	X	X		X	X	X	X	X
27.90	* Hexacosane	630-01-3	57, 71, 85	I	_	X	X	X	X	X	X		X
28.73	* Heptacosane	593-49-7	57, 71, 85	I	_	X		X	X	X	X	X	X
29.53	* Octocosane	630-02-4	57, 71, 85	I	_	X		X	X	X	X	X	X
29.64	* Squalene	111-02-4	69, 81	I	_	X	X	X	X	X	X	X	X
30.30	* n-Nonacosane	630-03-5	57, 71, 85	I	_	X		X			X	X	X
30.57	* Glycerol tricaprylate	538-23-8	127, 57, 327, 201	I	_	X						X	X

* Confirmed with standards.

A: Additive, M: Monomer.

** The restriction applies for the sum of a group of substances.

_: not included in European Union positive list.

**Table 3:** Compounds identified in the paper-based candy wrappers and their class of toxicity according to Cramer rules.

*t*_R_ (min)	Compound	CAS	*m*/*z*	TC	M1.1	M1.2	M2.1	M2.2	M3	M4	M5	PA.1	PA.2
13.81	* Triacetin	102-76-1	43, 103, 145	I	X	X	X	X	X	X	X		
16.21	* Butylated hydroxytoluene	128-37-0	205, 220	II			X	X	X	X	X		
17.35	* Diethyl phthalate	84-66-2	149, 177	I	X	X	X	X	X	X	X	X	
19.95	* Octadecane	593-45-3	57, 71, 85	I	X	X			X	X	X		
20.66	* Diisobutyl phthalate	84-69-5	149, 223	I	X	X	X	X	X		X	X	X
21.11	* Nonadecane	629-92-5	57, 71, 85	I	X	X	X	X	X	X	X		
21.18	7,9-Di-tert-butyl-1-oxaspiro(4,5)deca-6-9-diene-2,8-dione	82304-66-3	57, 205, 55, 175	III	X	X	X	X	X	X	X		
21.80	Palmitic acid	57-10-3	73,43, 129	I	X	X	X	X		X	X	X	
22.20	* Eicosane	112-95-8	57, 71, 85	I	X	X	X	X	X	X	X	X	
23.26	* Heneicosane	629-94-7	57, 71, 85	I	X	X	X	X	X	X	X		
23.66	Oleic acid	112-80-1	55, 97, 69, 83	I	X	X	X	X		X	X		
23.81	Tributyl aconitate	7568-58-3	112, 157, 139	I	X	X	X	X	X	X	X		
23.88	Octadecanoic acid (STEARIC ACID)	57-11-4	43,73, 60, 129	I	X	X	X	X		X	X		
24.25	* Docosane	629-97-0	57, 71, 85	I	X		X	X	X	X	X		
24.71	* Acetyl tributyl citrate	77-90-7	185, 259, 129	I	X		X	X	X	X	X	X	X
25.23	* Tricosane	638-67-5	57, 71, 85	I	X	X	X	X	X	X	X	X	X
25.69	* Benzyl butyl phthalate	85-68-7	91, 149, 206	I	X	X					X		
26.05	* Bis(2-ethylhexyl) adipate	103-23-1	129, 147	I	X	X			X				X
26.16	* Tetracosane	646-31-1	57, 71, 85	I	X	x	X	X	X	X	X	X	X
27.05	* Pentacosane	629-99-2	57, 71, 85	I		X	X	X	X		X		X
27.32	* Bis(2-ethylhexyl) phthalate	117-81-7	149, 167	I	X	X	X	X	X	X	X	X	X
27.91	* Hexacosane	630-01-3	57, 71, 85	I	X	X	X	X	X	X	X	X	
28.74	* Heptacosane	593-49-7	57, 71, 85	I	X	X	X	X	X	X	X		
29.12	2,4-dimethylicosane	75163-98-3	85, 84, 43, 57	I	X	X			X	X	X		
29.57	Heptadecylcyclohexane	19781-73-8	57, 82, 83	I	X	X	X		X	X	X		
29.64	* Squalene	111-02-4	69, 81	I	X	X	X	X	X		X	X	X
30.57	* Glycerol tricaprylate	538-23-8	127, 57, 327, 201	I								X	X
32.36	1,2-Didodecanoylglycerol	17598-94-6	183, 257	I	X	X				X	X		

* Confirmed with standards.
